# The value of serum amylase and drain fluid amylase to predict postoperative pancreatic fistula after pancreatoduodenectomy: a retrospective cohort study

**DOI:** 10.1007/s00423-021-02192-y

**Published:** 2021-05-14

**Authors:** Jelle C. van Dongen, Steven Merkens, M. Hossein Aziz, Bas Groot Koerkamp, Casper H. J. van Eijck

**Affiliations:** 1 Department of Surgery, Erasmus MC Cancer Institute, Rotterdam, The Netherlands; 2 Erasmus University Medical Center, Rg-231, Doctor Molewaterplein 40, 3015 GD Rotterdam, The Netherlands

**Keywords:** Pancreatoduodenectomy, Amylase, Pancreatic fistula

## Abstract

**Purpose:**

Serum and drain amylase have been identified as important predictors of postoperative pancreatic fistula (POPF) and might be useful to guide postoperative drain management after pancreatoduodenectomy. We aimed to determine and compare the value of serum amylase and drain fluid amylase to predict postoperative pancreatic fistula after pancreatoduodenectomy.

**Methods:**

This retrospective cohort study included patients after pancreatoduodenectomy from 2012 to 2019. The primary endpoint of our study was grade B/C POPF. Serum amylase on postoperative day 1 (SA-1) and drain fluid amylase on postoperative day 2 (DFA-2) were analyzed.

**Results:**

A total of 92 of 437 patients (21.1%) developed a grade B/C POPF. SA-1 was higher in patients who developed a grade B/C POPF (336 U/L vs. 97 U/L, p<0.001). Similarly, DFA-2 was higher in patients who developed a grade B/C POPF (1764 U/L vs. 78 U/L, p<0.001). SA-1 and DFA-2 had similar predictive accuracy (AUC: 0.82 vs. 0.85, respectively, p=0.329). Patients with SA-1<100 U/L (n=178) had a risk of 2.2% of developing grade B/C POPF, compared to 38.2% in patients with SA-1 >100 U/L (n=207). Patients with DFA-2<100 U/L (n=141) had a risk of 0% of developing grade B/C POPF, compared to 36.2% in patients with DFA-2>100 U/L (n=196). SA-1 and DFA-2 were strongly associated at a cut-off of 100 U/L (p<0.001, 89% concordance rate).

**Conclusion:**

Postoperative serum and drain amylase values below 100 U/L both effectively rule out POPF after pancreatoduodenectomy. The advantage of serum amylase measurement is that it can be used in patients who are managed without surgical drains.

**Supplementary Information:**

The online version contains supplementary material available at 10.1007/s00423-021-02192-y.

## Introduction

Postoperative pancreatic fistula (POPF) is the major determinant of morbidity after pancreatoduodenectomy [1, 2]. POPF can result in hemorrhage, abdominal sepsis, multisystem organ failure, and death [1, 3]. In an attempt to mitigate the effects of POPF, the intra-operative placement of intraperitoneal drains is commonly done, even though still controversial [4–6]. Studies suggest that early drain removal (≤ postoperative day (POD) 3) in low risk patients is associated with a decreased incidence of POPF and abdominal complications and reduced healthcare utilization costs compared to late drain removal (after POD 3) [7–9]. However, in current practice, a wide variation in postoperative drain management still exists [9].

Recently, postoperative serum amylase (SA) demonstrated to be an adequate predictor of POPF [10–14]. Therefore, SA might be useful in the guidance of drain removal. The removal of postoperative drains is generally based on the drain fluid amylase (DFA) output [7–9, 15–18]. Recent studies advocate a DFA threshold of 90–100 U/L for drain removal [16–18], remarkably lower than the 5000 U/L used in a prior randomized controlled trial [7]. Moreover, combining SA and DFA might improve prediction of POPF; however, this has only been studied in small series [12, 19]. Finally, SA can also predict POPF in patients who are managed without surgical drains.

Therefore, we aimed to determine and compare the value of serum amylase and drain fluid amylase to predict postoperative pancreatic fistula after pancreatoduodenectomy. Also, we aimed to establish clinically useful threshold values for serum amylase and drain fluid amylase.

## Methods

The Medical Ethical Review Committee of the Erasmus MC in Rotterdam, the Netherlands, approved this study and waived the need for informed consent (MEC-2018-1176).

### Study population

This retrospective cohort study included consecutive patients who underwent a pancreatoduodenectomy in the Erasmus MC from January 2012 to December 2019. Patients with additional concurrent resection, such as partial liver resections and hemicolectomies, were excluded.

### Data collection

Demographics, clinical characteristic, laboratory data, and operation details were extracted from prospectively maintained databases or from systematically reviewed patient charts. The diameter of the pancreatic duct was measured on preoperative computed tomography (CT) scan at the line of pancreatic transection anterior to the portal vein. Pancreatic texture was determined intra-operatively by the surgeon (soft/normal or hard). High-risk pathology was defined as anything other than pancreatic adenocarcinoma or chronic pancreatitis [1].

### Surgical and postoperative procedures

Blood serum laboratory measurements were routinely performed on POD 1, 3, and 5. SA was collected on POD 1 (SA-1) and POD 3 (SA-3) and DFA on POD 2 (DFA-2). The reference value of SA in our center was 0–52 U/L. In case of two DFA-2 values, the highest value was collected.

Both pylorus-ring resection and pylorus-preserving pancreatoduodenectomy were performed. The standard method of reconstruction was a pancreaticojejunostomy with duct-to-mucosa reconstruction. Pancreatic stents were not routinely placed. Intra-abdominal drains were routinely placed intra-operatively. Non-vacuuming silicon drains were placed in the proximity of the pancreaticojejunostomy and/or hepaticojejunostomy, most commonly dorsal of these anastomoses in the sub-hepatic space. Early postoperative drain removal was generally based on DFA and bilirubin levels on POD 2.

### Surgical outcomes

The primary endpoint of this study was the incidence of grade B/C POPF according to the 2016 International Study Group for Pancreatic Fistula definition [20]. Other surgical outcomes included delayed gastric emptying [21], post-pancreatectomy hemorrhage [22], and major complications defined as grade ≥ 3a according to the Clavien-Dindo classification [23].

### Statistical analysis

Continuous variables were expressed as a mean ± standard deviation or as a median ± interquartile range, depending on their distribution. For univariable analysis, continuous variables were compared using a T-test or a Mann-Whitney U test. Categorical variables were assessed using the chi-squared test or Fisher’s exact test (when a category includes < 5 patients). Diagnostic properties were determined based on receiver operating characteristic (ROC) curves. Based on the poor diagnostic value of SA-3, further analyses were limited to SA-1 and DFA-2. In the subset of patients with elevated SA-1 and DFA-2, univariable logistic regression was performed to determine odds ratios. A flowchart was constructed to evaluate the clinical value of combining SA-1 and DFA-2. Two-sided p-values < 0.05 were considered statistically significant. R statistical software (version 3.4.3; www.r-project.org) was used for all statistical analyses.

## Results

### Study population

The cohort consisted of 437 patients who underwent a pancreatoduodenectomy. Twenty patients were excluded due to concurrent other organ resections. In 357 patients (81.7%), a pancreatoduodenectomy with pylorus-ring resection was performed and in 80 patients (18.3%) a pylorus-preserving pancreatoduodenectomy. Thirty-three patients (7.6%) underwent preoperative chemoradiotherapy, and 13 patients (3.0%) underwent chemotherapy. Thirteen patients (3.0%) did not have a drain placed intra-operatively.

The most common pathological diagnoses included 169 pancreatic adenocarcinoma (39.0%), 51 distal cholangiocarcinoma (12.0%), 41 intraductal papillary mucinous neoplasms (IPMN) (9.4%), and 26 duodenal cancer (6.0%). Ninety-two patients (21.1%) developed a grade B/C POPF.

In univariable analysis, grade B/C POPF was associated with diabetes mellitus, neoadjuvant therapy, high-risk pathology, diameter of pancreatic duct, pancreatic texture, and intra-operative blood loss (Table 1).

**Table 1:** Patient characteristics stratified by postoperative pancreatic fistula^†^

	Overall (n = 437)	No POPF (n = 345)	POPF^†^ (n = 92)	p-value
Age	68 (59–73)	68 (59–73)	68 (58–75)	0.429
Male sex	244 (56%)	189 (55%)	55 (60%)	0.391
BMI	24.5 (22.3–26.9)	24.3 (22.3–26.6)	25.1 (22.4–27.7)	0.068
ASA status 3–4	119 (27%)	99 (29%)	20 (22%)	0.169
Diabetes mellitus	113 (26%)	100 (29%)	13 (14%)	0.004
Neoadjuvant therapy	47 (11%)	44 (13%)	3 (3.3%)	0.009
Type neoadjuvant therapy				0.016
No neoadjuvant therapy	390 (89%)	301 (87%)	89 (97%)	
Chemoradiotherapy	33 (7.6%)	32 (9.3%)	1 (1.1%)	
Chemotherapy	13 (3.0%)	11 (3.2%)	2 (2.2%)	
Radiotherapy	1 (0.2%)	1 (0.3%)	0 (0%)	
High-risk pathology^‡^	243 (56%)	166 (48%)	77 (84%)	<0.001
Malignant pathology	333 (76%)	263 (76%)	70 (76%)	0.941
Preoperative biliary drainage	267 (63%)	214 (64%)	53 (58%)	0.341
Pancreatic duct diameter (mm)	3 (2.0–6.0)	4 (2–6)	2 (1–4)	<0.001
Robot-assisted procedure	100 (23%)	75 (22%)	25 (27%)	0.270
Soft/normal pancreatic texture	150 (47%)	95 (38%)	55 (79%)	<0.001
Intra-operative blood loss (ml)	730 (400–1300)	700 (400–1300)	964 (500–1500)	0.044
CRP on POD 3 (mg/L)	206 (121–294)	186 (109–258)	307 (214–352)	<0.001
Days to drain removal in days	4 (3–6)	3 (3–5)	5 (3–18)	<0.001
Length of hospital stay	13 (9–23)	11 (8–16.5)	31 (20.5–53)	<0.001
Major morbidity (Clavien-Dindo ≥ grade 3)	173 (40%)	87 (25%)	86 (93%)	<0.001
Postoperative mortality	16 (3.7%)	8 (2.3%)	8 (8.7%)	0.008
Delayed gastric emptying grade B/C^¶^	104 (24%)	51 (15%)	53 (58%)	<0.001
Postpancreatectomy hemorrhage grade B/C^¥^	39 (8.9%)	15 (4.3%)	24 (26%)	<0.001
Readmittance due to surgical complications	75 (17%)	52 (15%)	23 (26%)	0.018

^†^Grade B/C postoperative pancreatic fistula according to the International Study Group for Pancreatic Surgery criteria

^‡^High-risk pathology included all pathological diagnosis except pancreatic ductal adenocarcinoma and chronic pancreatitis

^¶^Grade B/C delayed gastric emptying according to the International Study Group for Pancreatic Surgery criteria

^¥^Grade B/C post-pancreatectomy hemorrhage according to the International Study Group for Pancreatic Surgery criteria

*POPF* postoperative pancreatic fistula, *CRP* C-reactive protein, *POD* postoperative day

### Serum amylase and drain fluid amylase

SA-1 was available in 385 patients (88.1%) and SA-3 in 340 patients (77.8%). Median SA-1 was significantly higher in patients who developed POPF compared to patients who did not develop POPF (336 U/L vs. 97 U/L, p<0.001), with an AUC of 0.82 (95% CI: 0.78–0.86, p<0.001). Median SA-3 was also significantly higher in patients who developed POPF (55 U/L vs. 25 U/L, p<0.001), with an AUC of 0.75 (95% CI: 0.70–0.81). A higher risk of POPF is observed over the increasing quartiles of SA-1 (1.0% vs. 4.2% vs. 36.5% vs. 44.7%, respectively).

DFA-2 was available in 338 patients (77.3%). Median DFA-2 was significantly higher in patients who developed POPF compared to patients who did not develop POPF (1764 U/L vs. 78 U/L, p<0.001), with an AUC of 0.85 (95% CI: 0.81–0.89, p<0.001). The AUCs of SA-1 and DFA-2 did not differ significantly (p=0.329). Similarly, DFA-2 shows a rising incidence of POPF over the increasing quartiles (0% vs. 9.5% vs. 25.0% vs. 50.0%, respectively). No differences were observed between patients with and without available SA-1 or DFA-2 (Supplemental table 1). Both biomarkers showed a non-linear relationship with POPF (Supplemental figure 1).

Table 2 shows the median SA-1 and DFA-2 across patient characteristics. SA-1 and DFA-2 were both associated with preoperative chemoradiotherapy, diameter of the pancreatic duct, pancreatic texture, high-risk pathology (i.e., other than pancreatic cancer or chronic pancreatitis), and malignant pathology.

**Table 2:** Median serum amylase on postoperative day 1 and median drain fluid amylase on postoperative day 2 stratified by patient characteristics

	Median (IQR) serum amylase on POD 1 (U/L)	p-value	Median (IQR) drain amylase on POD 2 (U/L)	p-value
Age				
< 70	121 (50–335)	0.682	162 (33–1304)	0.753
≥ 70	117 (56–322)		163 (35–803)	
Gender				
Male	128 (56–308)	0.481	160 (36–1062)	0.494
Female	128 (56–308)		190 (34–1498)	
Body mass index				
< 25	103 (49–310)	0.113	114 (27–706)	<0.001
≥ 25	142 (56–345)		270 (61–1787)	
ASA score				
1–2	125 (53–337)	0.521	179 (35–1473)	0.362
3–4	105 (50–286)		134 (34–561)	
Preoperative biliary drainage				
No	160 (56–364)	0.203	270 (44–1584)	0.051
Yes	101 (52–293)		125 (32–1090)	
Preoperative chemoradiotherapy				
No	142 (57–354)	<0.001	221 (41–1420)	<0.001
Yes	55 (28–76)		20 (12–67)	
Diameter pancreatic duct				
≤ 3 mm	237 (79–502)	<0.001	528 (104–2086)	<0.001
> 3mm	72 (36–163)		49 (20–270)	
Pancreatic texture				
Soft/normal	258 (111–545)	<0.001	733 (162–2767)	<0.001
Hard/fibrotic	61 (31–120)		39 (20–155)	
High-risk pathology^†^				
No	73 (36–142)	<0.001	54 (21–253)	<0.001
Yes	220 (71–467)		454 (76–1914)	
Malignant pathology				
No	212 (75–509)	<0.001	672 (63–1996)	0.004
Yes	93 (49–270)		133 (32–769)	
Intra-operative blood loss				
≤ 1500 ml	115 (48–315)	0.038	162 (33–1231)	0.873
> 1500 ml	140 (61–429)		175 (35–989)	

^†^High-risk pathology included all pathological diagnosis except pancreatic ductal adenocarcinoma and chronic pancreatitis

*POD* postoperative day, *IQR* interquartile range

**Fig. 1:**
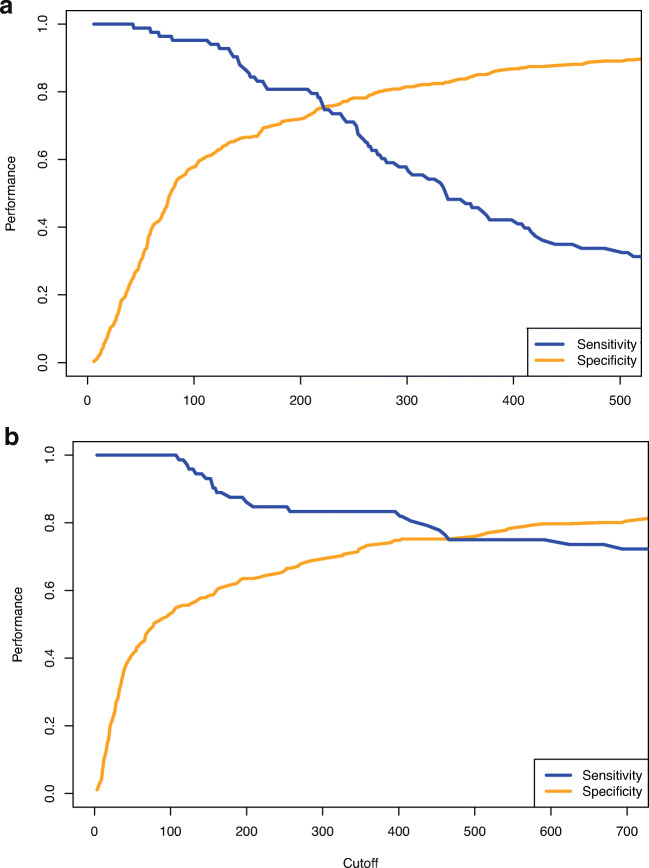
**a** Sensitivity and specificity of serum amylase on postoperative day 1. **b** Sensitivity and specificity of drain fluid amylase on postoperative day 2

Figure 1 displays the diagnostic properties of SA-1 and DFA-2 at different thresholds. Sensitivity remains close to 100% for both SA-1 and DFA-2 up to a cut-off value of 100 U/L. Patients with SA-1 < 100 U/L (n= 178) had a 2.2% risk on grade B/C POPF, compared to 38.2% in patients with SA-1 > 100 U/L (n= 207). The four patients with POPF and SA-1 <100 U/L experienced a grade B POPF due to radiological drainage of an amylase-rich fluid collection. Likewise, patients with DFA-2 < 100 U/L (n= 141) had a 0% risk on grade B/C POPF, compared to 36.2% in patients with DFA-2 > 100 U/L (n= 196). Patients with elevated SA-1 or DFA-2 experienced more postoperative complications had a longer length of hospital stay, and drains were removed later (Table 3).

**Table 3:** Surgical outcomes of patients by serum amylase on postoperative day 1 and drain fluid amylase on postoperative day 2

	SA-1 <100 N = 178	SA-1 >100 N = 207	p-value	DFA-2 <100 N = 141	DFA-2 >100 N = 197	p-value
Intra-operative blood loss	685 (315–1162)	800.0 (500–1400)	0.045	700 (400–1300)	730.0 (400–1375)	0.869
C-reactive protein (mg/L) on postoperative day 3	159 (99–246)	244 (158–325)	<0.001	141 (88–228)	231 (151–316)	<0.001
Days to drain removal in days	3 (3–4)	4 (3–7)	<0.001	3 (3–5)	4 (3–6)	0.004
Length of hospital stay	11 (8–17)	16 (10–31)	<0.001	11 (8–18)	15 (10–30)	<0.001
Major morbidity (Clavien-Dindo > grade 2)	43 (24%)	107 (52%)	<0.001	36 (26%)	93 (47%)	<0.001
Postoperative mortality	3 (1.7%)	9 (4.3%)	0.134	4 (2.8%)	4 (2.0%)	0.724
Postoperative pancreatic fistula^†^	4 (2.2%)	79 (38%)	<0.001	0 (0%)	72 (37%)	<0.001
Delayed gastric emptying grade B/C^¶^	27 (15%)	65 (31%)	<0.001	19 (13%)	63 (32%)	<0.001
Postpancreatectomy hemorrhage grade B/C^¥^	5 (2.8%)	25 (12%)	<0.001	4 (2.8%)	26 (13%)	<0.001
Readmittance due to surgical complications	29 (16%)	38 (19%)	0.549	22 (16%)	39 (20%)	0.306

^†^Grade B/C postoperative pancreatic fistula according to the International Study Group for Pancreatic Surgery criteria

^¶^Grade B/C delayed gastric emptying according to the International Study Group for Pancreatic Surgery criteria

^¥^Grade B/C post-pancreatectomy hemorrhage according to the International Study Group for Pancreatic Surgery criteria

In the subset of patients with SA-1 and DFA-2 > 100 U/L (n = 154), only intra-operative blood loss (p=0.006) and C-reactive protein (CRP) on POD 3 (p<0.001) were associated with POPF in univariable analysis (Supplemental table 2). CRP on POD 3 demonstrated an area-under-the-curve of 0.737 (0.652–0.822) in patients with SA-1 and DFA-2 > 100 U/L.

**Fig. 2:**
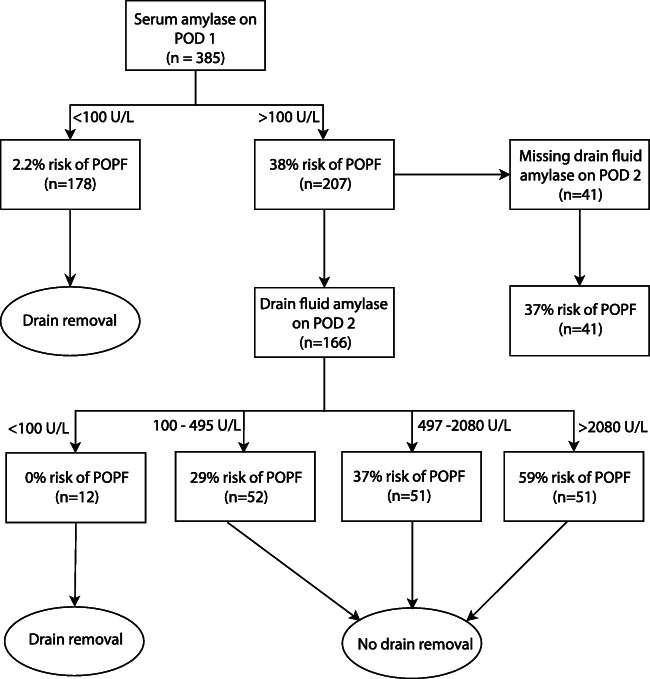
Flowchart of patients based on serum amylase on postoperative day 1 and drain fluid amylase on postoperative day 2

SA-1 and DFA-2 at a cut-off of 100 U/L were strongly associated (p<0.001), as there was concordance between the two parameters in 89% of the patients. Of patients with SA-1 <100 U/L, 113 (84.3%) also had a DFA-2 <100 U/L. A flowchart (Fig. 2) was constructed using a SA-1 threshold of 100 U/L. The patients with SA-1 >100 U/L were split in four groups based on their DFA-2 level. Twelve patients (7.2%) had a DFA-2 < 100 U/L. All remaining patients had a risk of more than 28% of developing POPF.

## Discussion

In this study, serum amylase on postoperative day 1 and drain fluid amylase on postoperative day 2 both effectively rule out grade B/C postoperative pancreatic fistula. Both biomarkers had a high negative predictive value, allowing for safe early drain removal in a subset of the patients. Based on our data, we propose a threshold of 100 U/L for both SA-1 and DFA-2. DFA-2 demonstrated little clinical value in addition to SA-1 in the identification of patients eligible for early removal of postoperative drains due to the strong relation between the two biomarkers.

Postoperative pancreatitis, defined as serum hyperamylasemia, has proven to be an important determinant in the development of POPF [10–14]. It is hypothesized that postoperative inflammation of pancreatic tissue causes impaired healing of the pancreatojejunal anastomosis, resulting in leakage of enzymatic fluid in the abdominal cavity. We found that patients with POPF had higher CRP levels on POD 3, indeed suggesting early inflammatory processes [24]. Another hypothesis is that the peritoneal membrane reuptakes leaked amylase-rich fluid from the abdominal cavity increasing serum amylase levels. The absence of serum hyperamylasemia (< 100 U/L) was associated with a 2.2% risk of developing POPF, compared to 38.2% in patients with SA-1 > 100 U/L (PPV = 38.2%, NPV = 97.8%). Similarly, a recent retrospective study demonstrated a high negative predictive value for SA using Connor’s cut-off for SA (SA < 50 U/L, PPV = 36.2%, NPV = 95.3%) and the Atlanta criteria (SA < 150 U/L, PPV = 53.8%, NPV = 88.2%) in 292 patients [14]. In addition, Palani velu et al. recommended an optimal threshold of SA on the night after pancreatoduodenectomy of 130 U/L (PPV= 36.7%, NPV=88.8%) in 185 patients [11]. Kuhlbrey et al. also demonstrated a high negative predictive value of SA-1 < 159 U/L (PPV = 48%, NPV =92.2%) in 561 patients after pancreatoduodenectomy.[13] Based on our data, a threshold of 100 U/L for safe drain removal is optimal on POD 1.

Drain fluid amylase is a well-established predictor of POPF [15–18]. The randomized controlled trial (RCT) by Bassi et al. demonstrated that early drain removal (POD 3) was associated with a reduced rate of postoperative complications compared to late drain removal (POD 5 or beyond) [7]. In this trial, drain removal was based on a threshold value of DFA < 5000 U/L on POD 1; however, our study suggests that a lower threshold might be more suitable. Patients with DFA-2 < 100 U/L had a 0% chance on POPF, compared to 36.2% in patients with DFA-2 >100 U/L. Elevating the threshold level led to a marginal increase in the positive predictive value, while the negative predictive value decreased substantially. Likewise, Lee et al. demonstrated in 380 patients after pancreatoduodenectomy that patients with DFA-1 < 90 U/L had a 2.1% chance of POPF. [16] A recent meta-analysis also demonstrated that at a threshold of DFA-1 < 100 U/L patients had a 3% risk of developing POPF, also suggesting that a low DFA threshold should be used for selective drain removal [18].

However, only few small studies compared the value of serum amylase and drain fluid amylase in the early postoperative setting. An advantage of SA is that it can be measured in patients without surgical drains. The need for intra-operative drain placement after pancreatoduodenectomy remains debated. Three RCTs have investigated the need for surgical drainage after pancreatoduodenectomy: two RCTs found fewer complications without drains [4, 6], and one RCT was discontinued because of increased mortality in patients without drains [5]. Early drain removal has proven to lower POPF and abdominal complication rates in patients at low risk of developing POPF after pancreatoduodenectomy [7, 8]. Given the high negative predictive value of SA-1 and DFA-2, both may facilitate the selective removal of drains in patients at low risk of developing POPF. Early drain removal guided by SA might be suited to substitute DFA in practice. Blood is routinely drawn after pancreatoduodenectomy, thereby SA is an easily obtainable marker to guide drain management. Nonetheless, our data suggest that follow-up of patients at high risk of developing POPF using SA is not feasible, as it quickly normalizes in patients with elevated SA-1.

A limitation of using SA-1 at a threshold of 100 U/L is that in approximately half of the patients, postoperative drains will be left in place. These patients would require closer monitoring with a low threshold for a CT in case of clinical suspicion of pancreatic leakage or infected fluid collections. Other biomarkers and clinical parameters should be used to identify patients requiring additional CT scan imaging. For example, CRP values may facilitate the identification of patients with an elevated risk of POPF after pancreatoduodenectomy on POD 3 and 5 [24].

Diameter of the pancreatic duct, pancreatic texture, intra-operative blood loss, and other diagnoses than pancreatic adenocarcinoma or chronic pancreatitis are well-known risk factors for POPF [1, 25]. Risk scores based on these parameters are widely applied in clinical practice to estimate the risk of developing POPF in patients, such as the fistula risk score [1, 25]. These risk factors showed limited predictive value in combination with SA-1 and DFA-2. In the subset of patients with SA-1 and DFA-2 > 100 U/L, only BMI and intra-operative blood loss were associated with POPF. Hence, SA-1 and DFA-2 could rather be used in the early postoperative setting to identify patients at high risk of POPF.

We present one of the first studies to demonstrate that the combination of SA-1 and DFA-2 might hold little additional clinical value in the early removal of drains. However, our study has certain limitations. First, due to the retrospective nature of our study, we had missing SA-1 and DFA-2 values. Furthermore, patients with high SA-1 or DFA-2 might be subjected to later drain removal and closer monitoring, which might introduce verification bias. However, generally interventions are not based on SA-1 or DFA-2 values, but rather on clinical and imaging characteristic later in the postoperative course. Finally, some of the POPF in patients with mildly elevated drain amylase may have developed because the drain was not removed, as suggested by the RCT of Bassi et al. [7].

In future research, our findings should be externally validated to analyze the generalizability of our results. Also, the feasibility of early drain removal and postoperative monitoring based on serum amylase values should be evaluated in well-designed prospective cohort studies.

## Conclusion

SA-1 and DFA-2 values < 100 U/L seem to effectively rule out POPF and could be useful to guide the early removal of intra-operatively placed drains. The combination of SA-1 and DFA-2 might not facilitate early drain removal in more patients. An advantage of serum amylase measurement is that it does not require the placement of surgical.

## Supplementary Information

ESM 1 (PDF 195 kb)

ESM 2 (PDF 135 kb)

ESM 3 (PDF 233 kb)
