# Supplementary material for: The value of serum amylase and drain fluid amylase to predict postoperative pancreatic fistula after pancreatoduodenectomy: a retrospective cohort study
Source: Langenbecks Arch Surg. 2021 May 14;406(7):2333–41. doi: 10.1007/s00423-021-02192-y (PMC8578085; doi:10.1007/s00423-021-02192-y)

Supplemental figure 1a: Linear and non-linear fitted log odds ratios for serum amylase on postoperative day 1

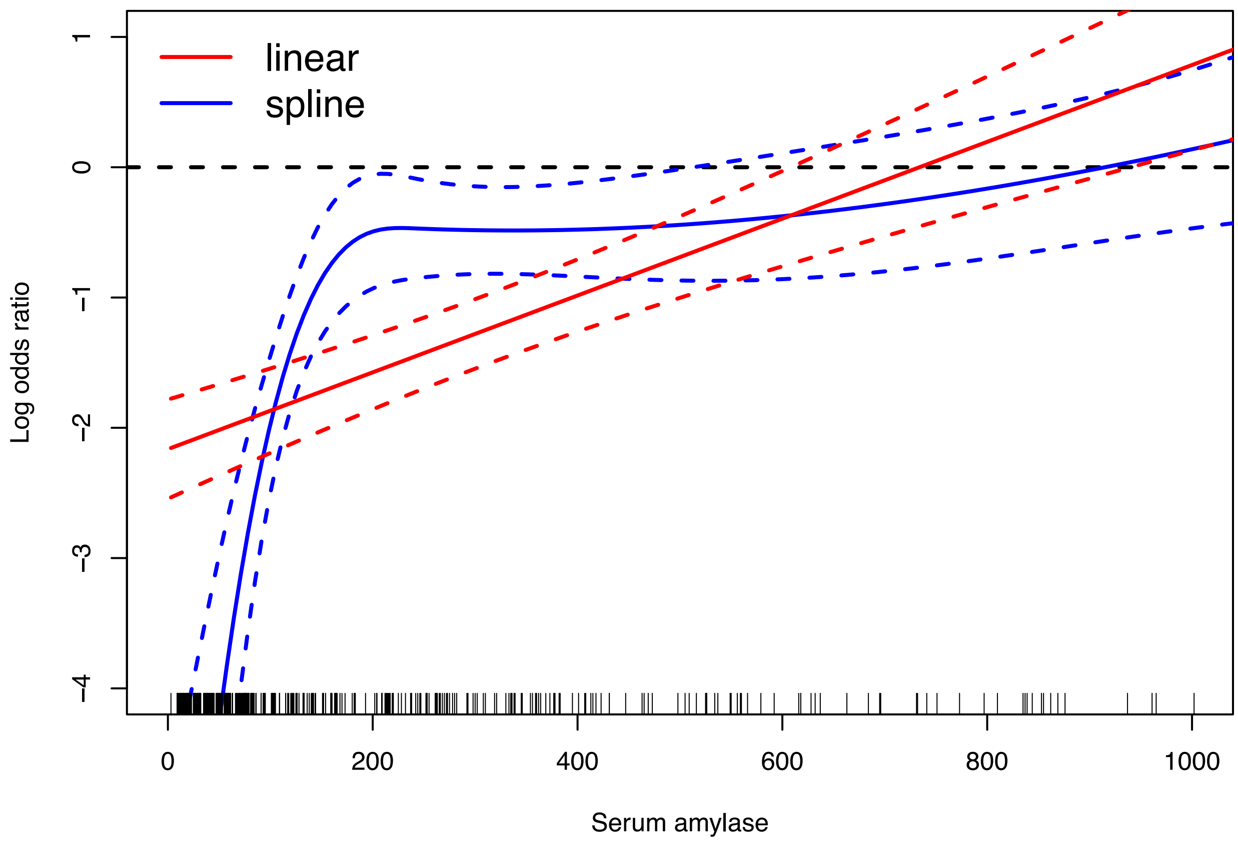

Supplemental figure 1b: Linear and non-linear fitted log odds ratios for drain fluid amylase on postoperative day 2

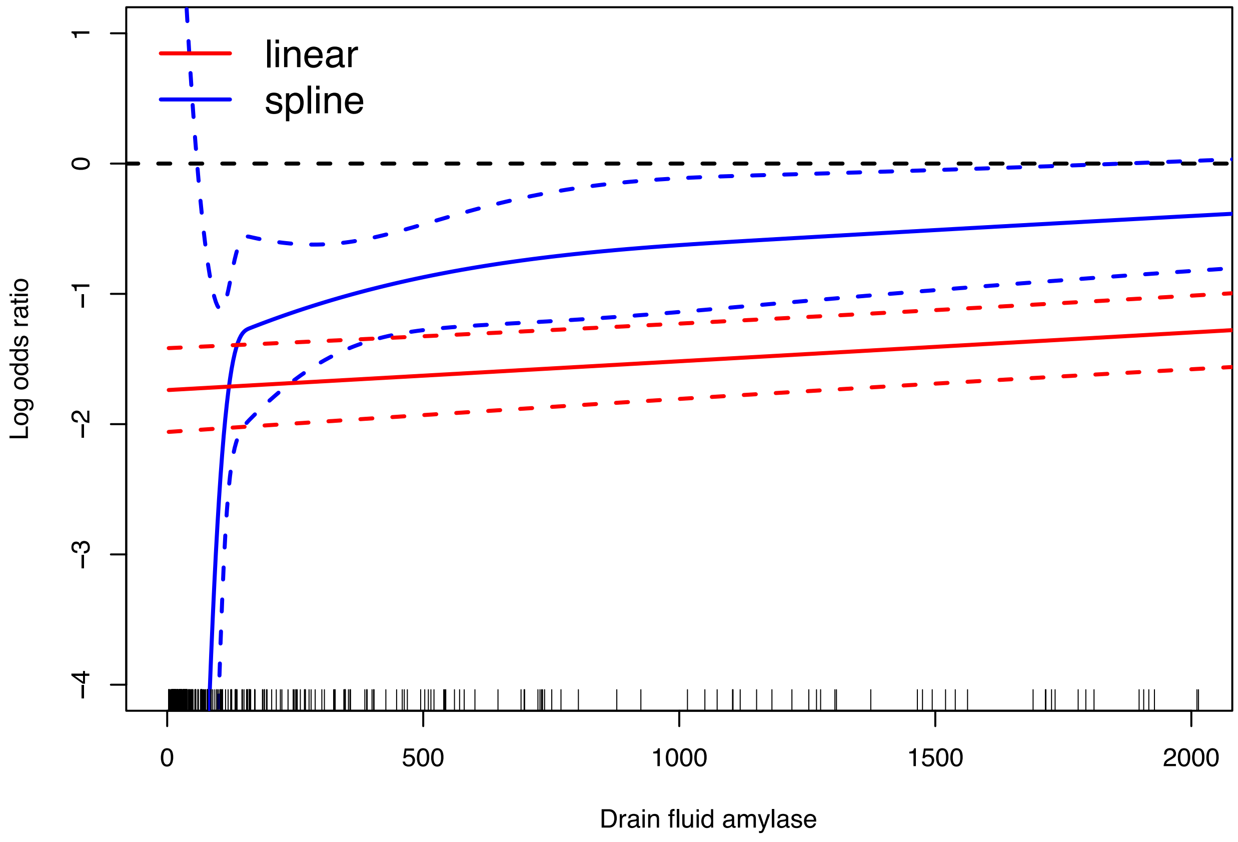

Supplement: Supplementary file 1 — (PDF 195 kb) [file 423_2021_2192_MOESM1_ESM.pdf]
