# Supplementary material for: The value of serum amylase and drain fluid amylase to predict postoperative pancreatic fistula after pancreatoduodenectomy: a retrospective cohort study
Source: Langenbecks Arch Surg. 2021 May 14;406(7):2333–41. doi: 10.1007/s00423-021-02192-y (PMC8578085; doi:10.1007/s00423-021-02192-y)
Supplement: Supplementary file 2 — (PDF 135 kb) [file 423_2021_2192_MOESM2_ESM.pdf]

Supplemental table 1A: Baseline characteristics of patients with missing serum amylase on postoperative day 1

	Missing serum amylase N = 52	Available serum amylase N = 385	p-value
Age	68.0 (58.5 - 72.9)	67.7 (59.0 - 73.0)	0.899
Male sex	27 (52%)	217 (56%)	0.504
BMI	24.2 (22.0 - 25.9)	24.5 (22.4 - 27.1)	0.367
ASA status 3-4	16 (31%)	103 (27%)	0.564
Diabetes Mellitus	16 (31%)	97 (25%)	0.389
Neoadjuvant therapy	3 (5.8%)	44 (11%)	0.216
Neoadjuvant therapy type			0.278
No	49 (94%)	341 (89%)	
Chemoradiotherapy	1 (1.9%)	32 (8.3%)	
Chemotherapy	2 (3.8%)	11 (2.9%)	
Radiotherapy	0 (0%)	1 (0.3%)	
High risk pathology	28 (54%)	215 (56%)	0.770
Malignant pathology	42 (81%)	291 (76%)	0.427
Preoperative biliary drainage	30 (61%)	237 (63%)	0.890
Pancreatic duct diameter	3.0 (2.0 - 5.0)	3.0 (2.0 - 6.0)	0.325
Robot-assisted procedure	16 (31%)	84 (22%)	0.149
Soft pancreatic texture	20 (53%)	130 (45%)	0.506

Supplemental table 1B: Baseline characteristics of patients with missing drain fluid amylase on postoperative day 2

	Missing drain fluid amylase N = 99	Available drain fluid amylase N = 338	p-value
Age	68.4 (60.0 - 73.0)	67.7 (58.4 - 73.0)	0.231
Male sex	53 (54%)	191 (57%)	0.600
BMI	24.6 (22.2 - 28.2)	24.4 (22.4 - 26.7)	0.292
ASA status 3-4	30 (30%)	89 (27%)	0.464
Diabetes Mellitus	26 (26%)	87 (26%)	0.917
Neoadjuvant therapy	12 (12%)	35 (10%)	0.618
Neoadjuvant therapy type			0.313
No	87 (88%)	303 (90%)	
Chemoradiotherapy	7 (7.1%)	26 (7.7%)	
Chemotherapy	4 (4.0%)	9 (2.7%)	
Radiotherapy	1 (1.0%)	0 (0%)	
High risk pathology	53 (54%)	190 (56%)	0.616
Malignant pathology	76 (77%)	257 (76%)	0.917
Preoperative biliary drainage	64 (67%)	203 (61%)	0.341
Pancreatic duct diameter	3.0 (2.0 - 5.0)	3.0 (2.0 - 6.0)	0.692
Robot-assisted procedure	22 (22%)	78 (23%)	0.859
Soft pancreatic texture	33 (45%)	117 (48%)	0.592
