# Supplementary material for: The value of serum amylase and drain fluid amylase to predict postoperative pancreatic fistula after pancreatoduodenectomy: a retrospective cohort study
Source: Langenbecks Arch Surg. 2021 May 14;406(7):2333–41. doi: 10.1007/s00423-021-02192-y (PMC8578085; doi:10.1007/s00423-021-02192-y)
Supplement: Supplementary file 3 — (PDF 233 kb) [file 423_2021_2192_MOESM3_ESM.pdf]

Supplemental table 2: Characteristics of patients with elevated SA-1 and DFA-2 (> 100 U/L) stratified by postoperative pancreatic fistula†

	**Overall (n = 154)**	**No POPF (n = 90)**	**POPF (n = 64)**	**p-value**
Age	67.2 (57.2 - 73.0)	66.8 (57.5 - 72.5)	67.7 (57.8 - 74.7)	0.325
Male sex	87 (56%)	46 (51%)	41 (64%)	0.110
BMI	24.5 (22.5 - 26.8)	24.1 (22.7 - 26.4)	25.1 (22.3 - 27.4)	0.173
ASA status 3-4	36 (24%)	22 (25%)	14 (22%)	0.682
Diabetes Mellitus	24 (16%)	16 (18%)	8 (12%)	0.374
Neoadjuvant therapy	6 (3.9%)	4 (4.4%)	2 (3.1%)	>0.999
Neoadjuvant therapy type				0.825
No	148 (96%)	86 (96%)	62 (97%)	
Chemoradiotherapy	2 (1.3%)	1 (1.1%)	1 (1.6%)	
Chemotherapy	4 (2.6%)	3 (3.3%)	1 (1.6%)	
Radiotherapy	0 (0%)	0 (0%)	0 (0%)	
High risk pathology	110 (72%)	60 (67%)	50 (78%)	0.146
Malignant pathology	104 (68%)	58 (65%)	46 (72%)	0.380
Preoperative biliary drainage	86 (57%)	50 (57%)	36 (57%)	0.968
Pancreatic duct diameter (mm)	2.0 (1.0 - 4.0)	3.0 (2.0 - 4.0)	2.0 (1.0 - 4.0)	0.121
Robot-assisted procedure	35 (23%)	17 (19%)	18 (28%)	0.178
Soft/normal pancreatic texture	81 (74%)	45 (73%)	36 (75%)	0.775
Intra-operative blood loss (ml)	725.0 (419 - 1325)	500 (400 - 1200)	1000 (500 - 1700)	0.006
CRP on POD 3 (mg/L)	245 (160 - 322)	207 (135 - 268)	313 (220 - 360)	<0.001

† Grade B/C postoperative pancreatic fistula according to the International Study Group for Pancreatic Surgery criteria.
‡ High risk pathology included all pathological diagnosis except pancreatic ductal adenocarcinoma and chronic pancreatitis
